# The complete mitochondrial genome of Yao silkworm (*Bombyx mori*)

**DOI:** 10.1080/23802359.2019.1660244

**Published:** 2019-09-06

**Authors:** Gui-Zheng Zhang, Wen-Gong Huang, Yu-Li Zhang, Yan-Wei Liu, Hua-Xu Huang, Yan-Qun Liu, Li-Hui Bi, Cheng Lu

**Affiliations:** a Guangxi Institute of Sericulture Science, Nanning, China;; b Department of Sericulture, College of Bioscience and Biotechnology, Shenyang Agricultural University, Shenyang, China;; c State Key Laboratory of Silkworm Genome Biology, Southwest University, Chongqing, China

**Keywords:** Yao silkworm, mitochondrial genome, phylogenetic relationship

## Abstract

Here, we describe the first complete mitochondrial genome of Yao silkworm, a unique silkworm resource native at Guangxi, China. This circular molecule is 15,656 bp long and contains the typical set of 37 genes (13 protein-coding genes, two ribosomal RNA genes, and 22 transfer RNA genes) and one non-coding A + T-rich region of 494 bp long. The genome organization and gene arrangement are identical to those observed in all available *Bombyx mori* strains. The phylogenetic tree inferred from Bayesian inference provides a molecular evidence that Yao silkworm belongs to the domestic silkworm (*B. mori*), rather than a novel silkworm species.

The white-trousers Yao nationality resided in Nandan County, Guangxi, China has been regarded as “the living fossil of human civilization” by the United Nations Educational, Scientific and Cultural Organization. The white-trousers Yao has self-propagated, self-reared and self-used Yao silkworm to produce flat plate silk for thousands of years, and the Yao silkworm has been regarded as the living fossil of Chinese silk culture (Lu et al. 2013). Yao silkworm is mainly distributed into 29 villages in Nandan County. Yao silkworm has characteristics with fine denier, univoltine, flat plate silk, natural golden yellow silk, and natural trimolter (Bai et al. 2015). In this study, we present the first mitochondrial genome of Yao silkworm, providing basic genetic information for this silkworm resource.

The inbred strain 17 W of Yao silkworm used in this study was initially collected from Nandan County (N25°10′21.53′′; E107°43′54.66′′), Guangxi, China, and then successively subcultured by Guangxi Institute of Sericulture Science (N22°50′43.31′′; E108°13′43.80′′), Nanning, China. A single pupa was used to extract the total DNA. The specimen and its DNA are stored with the archival number of Yao_17W in Guangxi Institute of Sericulture Science. Two over-lapping fragments of ∼8 kb were amplified with specific primers. Then, the amplified products were sequenced on Illumina Hiseq platform by Frasergen Co., Ltd., Wuhan, China. A reference-guided assembly was used to obtain the mitochondrial genome and *Bombyx mori* strain C108 (AB070264; Yukuhiro et al. 2002) as the reference. The genome was annotated with MITOS (Bernt et al. 2013) and manually corrected based on the known mitochondrial genomes of *Bombyx* species. The mitochondrial genome of Yao silkworm 17 W has been deposited in GenBank under accession no. MN027269.

The mitochondrial genome of 17 W is 15,656 bp in length and contains a typical gene complement of metazoan: 13 protein-coding genes (PCGs), 22 tRNA genes, two rRNA genes, and an A + T-rich region. The order and arrangement of this genome are identical to those of *Bombyx* species available. All protein-coding genes start with a typical ATN initiation codon, except for *COI* that begins with atypical codon CGA. Ten of 13 PCGs end with TAA, and the incomplete stop codons T or TA are found in *COI*, *COII*, and *NAD4L*. All 22 typical tRNA genes are observed in the mitochondrial genome of Yao silkworm. The A + T-rich region of 17 W spans 494 bp long, and harbors one copy of the repeat element of 126 bp, as found in all sequenced *B. mori* strains.

We built the phylogenetic relationship of Yao silkworm and the domestic silkworm using Bayesian inference with Mrbayes 3.1.2 (Huelsenbeck and Ronquist, 2001). The mitochondrial genome provided evidence that Yao silkworm belongs to the domestic silkworm (*B. mori*), rather than a novel silkworm species (Figure 1). The mitochondrial phylogeny of silkworms also suggested that Yao silkworm is a unique silkworm resource that need to be attached much importance to the protection and development.

**Figure 1.**
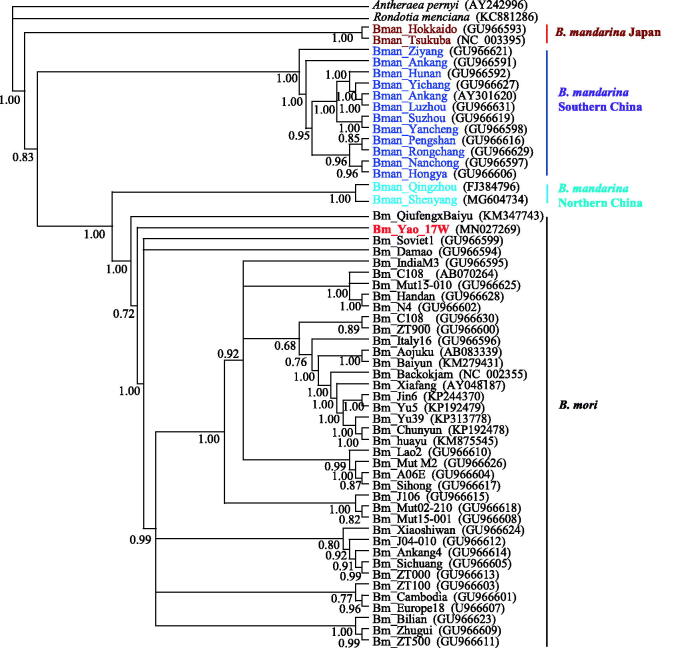
Phylogenetic relationship of *Bombyx* silkworms inferred from whole mitochondrial genome sequence using Bayesian inference with GTR + G + I model. Included are *B. mori*, Japanese *B. mandarina*, northern Chinese *B. mandarina*, and southern Chinese *B. mandarina*. *Rondotia menciana* (Bombycidae), and *Antheraea pernyi* (Saturniidae) serve as outgroups. The Markov chain Monte Carlo (MCMC) search was conducted for 2,000,000 generations, and sampling was done every 1000 generation. The final average standard deviation of split frequencies was 0.0050. The posterior probability values are indicated at the nodes. The accession numbers are listed following silkworm name.

## References

[CIT0001] Bai X, Zhang GZ, Huang MM, Wei SP, Li L, He ST 2015 Construction of Guangxi's special silk culture by means of minority silk culture. China Sericult. 36:77–81.

[CIT0002] Bernt M, Donath A, Juhling F, Externbrink F, Florentz C, Fritzsch G, Putz J, Middendorf M, Stadler P 2013 MITOS: improved de novo metazoan mitochondrial genome annotation. Mol Phylogenet Evol. 69:313–319. 22982435 10.1016/j.ympev.2012.08.023

[CIT0003] Huelsenbeck JP, Ronquist F 2001 MRBAYES: Bayesian inference of phylogenetic trees. Bioinformatics. 17:754–755. 11524383 10.1093/bioinformatics/17.8.754

[CIT0004] Lu WJ, Lu MM, Yu CJ, Qin QM 2013 Enlightenment of Baiku Yao silkworm and silk production on modern silkworm breeding. Guangdong Canye. 47:5–7.

[CIT0005] Yukuhiro K, Sezutsu H, Itoh M, Shimizu K, Banno Y 2002 Significant levels of sequence divergence and gene rearrangements have occurred between the mitochondrial genomes of the wild mulberry silkmoth, *Bombyx mandarina*, and its close relative, the domesticated silkmoth, *Bombyx mori*. Mol Biol Evol. 19:1385–1389. 12140251 10.1093/oxfordjournals.molbev.a004200

